# CDKN2B Methylation Correlates with Survival in AML Patients

**Published:** 2017

**Authors:** Esmat Kamali Dolatabadi, Mohammadreza Ostadali Dehaghi, Naser Amirizadeh, Kazem Parivar, Reza Mahdian

**Affiliations:** a *Department of Biology, Science and Research Branch´ Islamic Azad University, Tehran Iran. *; b *Cell Therapy and Hematopoietic Stem Cell Transplantation Research Center, Tehran University of Medical Sciences.Tehran, Iran. *; c *Iranian Blood Transfusion Research Center, High Institute for Research and Education in Transfusion Medicine, Tehran, Iran.*; d *Molecular Medicine Department, Pasteur Institute of Iran, Tehran, Iran.*

**Keywords:** Epigenetic, Methylation, AML, Melting curve analysis (MCA), Real-Time PCR

## Abstract

Aberrant DNA methylation has been reported as an important phenotype in acute myeloid leukemia. However the clinical significance of methylation changes has not been clear yet. In this study methylation Specific Melting Curve Analysis (MS-MCA) and real time PCR was used to assess the CDKN2B promoter hyper-methylation and gene expression in 59 Iranian acute myeloid leukemia (AML) patients. The incidence of aberrant hyper methylation of CDKN2B gene and cytogenetic abnormalities were 37.3% (22 of 59 patients) and 35.6% (21 of 59) respectively in our patients. We observed that CDKN2B expression level was lower than normal mesenchymal stem cells. Our data revealed significant correlation between methylated *CDKN2B* promoter region and mRNA gene expression (*P*= 0.007). Also, our data indicated that AML patients with aberrant methylation of CDKN2B gene had a lower survival rates (*P*=0.043). In addition, they had a higher proportion of leukemic blast cells (*P*=0.022) and higher white blood cell count in peripheral blood (*P*=0.0123). Aberrant methylation of CDKN2B was observed to higher in M2 subtype and lower in M3 and M4 subtypes. Although, we observed a significant correlation between Methylation and survival, there was no significant correlation between CDKN2B methylation and treatment outcome of AML patients (*P*=0.187). Furthermore, our data didn’t illustrated a significant correlation between CDKN2B expression and survival (*P*=0.93). In conclusion our study showed that the aberrant methylation is one of molecular mechanisms involved in CDKN2B gene expression, moreover we can consider the CDKN2B methylation, as a prognostic marker in predict AML patients’ survival.

## Introduction

Acute myeloid leukemia (AML) is a clonal hematopoietic disorder that may be derived from either a hematopoietic stem cell or a lineage-specific progenitor cell. Etiology of AML is unknown yet but, there are number of risk factors for the development of AML. Genetic predisposition (including Fanconi´s anemia, ataxia-telangiectasia,neurofibromatosis, Bloom´s syndrome, and Down´s syndrome), environmental exposure (ionizing radiation and organic solvents such as benzene), prior therapy, prior bone marrow disorders (aplastic anemia, paroxysmal nocturnal hemoglobinuria, and severe congenital neutropenia) and age are some of most important risk factor. In additional, mutations and epigenetic changes has been reported in AML too. Therapy for AML includes remission induction followed by post remission chemotherapy for most patients. For some, this is followed by hematopoietic stem cell transplantation (HSCT). Treatment recommendations for AML vary, taking into account patient age, cytogenetics, and prognostic factors. The recommendations are often divided into those for patients younger than 60 and those 60 years and older. AML patients younger than 60 years have complete response rates of 70% to 80% after induction chemotherapy. Overall survival, however, remains at only about 50% for those who go into a complete remission, or 30% to 40% overall. But overall survival in patients more than 60 years who goes to complete remission is 5-10% (1). 

As we mentioned, epigenetic changes, which do not affect the genetic code itself but which influence the activity of genes, play some roles in the pathogenesis of acute myeloid leukemia (2). Recent studies have showed that DNA methylation and histone modification are most important epigenetic changes in AML. Methylation of CpG islands reduces gene transcription and is purported to play a role in malignancy through reduced expression of tumor suppressors and genes concerned with differentiation. Global hypo methylation is also frequently observed in malignant cells, and while it is likely that there is genetic instability and promotion of proto oncogene expression, the exact role of global methylation patterns in the development of cancer is uncertain (3,4). The clinical responses of AML to drugs that reverse aberrant hyper methylation, such as 5-aza-2′-deoxycytidine and 5-azacytidine, suggest that aberrant hyper methylation plays a causative role in disease and is not just a side effect resulting from dysregulated proliferation or DNA damage. While genetic hits are fixed irreversible states of gene inactivation, epigenetic events do not interfere with the information content of the affected genes and epigenetic changes are potentially reversible (5,6). As we mentioned, methylation of the cytosine’s palindromic CpG sites which clustered in gene promoter regions plays an important role in the epigenetic silencing of genes which concerned in development, progression and relapse of leukemia such as *ESR1*, *IGSF4*, and *CDKN2B* (2, 4, 7). 

*CDKN2B* gene lies adjacent to the *CDKN2A* tumor suppressor gene in a region at 9p21 and this gene is frequently mutated and deleted in a wide variety of tumors (8,9). This gene encodes a cyclin-dependent kinase inhibitor, known as *CDKN2B* protein, which is a cell cycle regulator that inhibits cell cycle G1 progression (10). Although the methylation of the 5´ promoter region of genes, which leads to transcriptional silencing, is the major mechanism of *CDKN2B* gene inactivation in acute myeloid leukemia (AML), homozygous deletion or intragenic mutation mechanism of *CDKN2B* inactivation in AML was reported rarely (9-11). 

This study was done to investigate methylated *CDKN2B* gene as a diagnostic or prognostic biomarker in Iranian adult patients with AML. Furthermore, this study indicated the incidence of methylation in *CDKN2B* and correlation between *CDKN2B* methylation and gene expression. 

## Experimental

*AML Patients and Normal controls*

Bone marrow sample of untreated (de novo and secondary acute myeloid leukemia) AML patients and previously treated AML (secondary or relapsed AML) AML patients who referred to the Hematology Oncology and Stem Cell Transplantation Research Center Shariati hospital (HORCSCT) were collected during 6 month. Out of the 103 collected samples 59 of them were included in this study. Informed consent was obtained in accordance with the declaration of Hematology, Oncology and Stem Cell Transplantation Research Center Shariati hospital. Bone marrow and/or peripheral blood smear of all cases were studied and diagnosis­ was confirmed by flow cytometry and molecular analysis. AML patients were classified according FAB classification (12). In order to following up the methylation changes, sampling was done in half of induction and end of induction. All accessible patients were followed more than 1 year after partial/complete remission. 20 normal individuals with 17-56 years old were entered to our study as normal controls. 

*Drugs and therapy*

Standard induction chemotherapy including Cytarabine and Anthracycline for non-acute promyelocytic leukemia patients (non APL) or Arsenic Trioxide for acute promyelocytic leukemia patients (APL) was given aiming complete remission (CR) (13). 

*DNA extraction and Sodium bisulfite modification*

Mononuclear cells were isolated from the bone marrow of enrolled AML patients using Ficoll-Paque (Amersham Pharmacia Biotech, Uppsala, Sweden) and genomic DNA was extracted using DNA mini kit (Qiagen, Valencia, CA) according to instruction. DNA quality and concentration was determined by Nano Drop® ND-1000 spectrophotometer (Thermo scientific, USA).

Bisulfite treatment of genomic DNA was carried out using Lµg of DNA according to protocol provided by EpiTect bisulfite kit (Qiagen) 

*Primer design and Methylation Specific Melting Curve Analysis (MS-MCA)*

Primers were designed covering 14 CpG sites in CDKN2B promoter CpG Islands region by Meth Prime software. Details of primers sequences and genomic locations are given in Table 1.

Methylation Specific Melting Curve Analysis (MS-MCA) method was applied to analyze promoter methylation in all patients and controls (14). PCR reactions were carried out in a final volume of 25 µL containing 50ng of bisulfite modified DNA, 10 p.moL of each forward and reverse primes and 10 µL SYBR Premix EX Taq TM II (Takara, Japan) in 96 wells ABI plate. Reactions were run on an ABI StepOne Plus using the optimized parameters (95 °C, 2mins; 40 cycle of: 10 sec at 95 °C, 15 sec at 57 °C and 25 sec at 72 °C; melting curve: 10 sec at 95 °C, 60 sec at 60 °C and continues melting). The sensitivity and specificity of the assay were validated using Epitect methylation control set (QIAGEN, EpiTect). 

*CDKN2B Gene expression assay*

*RNA extraction, cDNA synthesis and Real time PCR*

Total RNA was isolated from 2×10^6^ MNCs of AML patients by Trizol method (Invitrogen, CA, USA). The integrity of RNA samples were determined using denaturing agarose gel electrophoresis and concentrations of the RNAs were determined by Nano Drop® ND-1000 spectrophotometer (Thermo scientific, USA).

The reverse transcription reaction was performed with first strand cDNA synthesis kit (Fermentase, #K1621) using random primer hexamers, according to the manufacturer’s instructions. 

*CDKN2B* and *GAPDH* primers (Table 1) were designed using primer express software v.3.0 (Applied Biosystems, CA, USA).

Mesenchymal Stem Cell (MSC) was used as normal control. MSCs were obtained from cord blood mononuclear cells by negative immune depletion of CD3+, CD14+, CD19+, CD38+, Cd66b+ and glycophorin A+ cells using a commercially available kit (RosetteSep, Stem Cell Technologies, Vancouver, BC, Canada). 

Real time PCR was performed for patients and mesenchymal Stem Cell (MSC) as normal control using the ABI StepOne real time thermo cycler and the SDS software v.1.2.3 (Applied Biosystems, CA, and USA). Reaction volume was 20 μL including 10 μL SYBR Premix EX Taq TM II (Takara, Japan), 10 p.m of each forward and reverse primers and cDNA. Thermal cycling was: 10 min at 95 ºC and followed by 40 cycles at 95 ºC for 15sec and 60 ºC for 1min. Glyceraldehyde-3-Phosphate Dehydrogenase (*GAPDH*) was used as internal reference gene, according to 2 ^-ΔΔCT^ method (15,16) using MSC cells as normal expression. 

*Statistical analysis*

Statistical analysis data was carried out in the Statistical Package for the Social Sciences software (SPSS 18). Demographic and baseline characteristics of patients’ cohort were compared using Pearson chi square and Fisher exact test. Wilcoxon test was used to investigate correlation between methylation and expression. Survival data was presented using the Kaplan-Meier and Log Rank (Mantel-Cox) test. P-value less than 0.05 consider as significant.

## Results

*Patients and clinical finding*

A total of 59 affected individuals (51 non APL individuals, 8 APL individual) with untreated new (47 individual), secondary (4 individuals) and relapsed (8 individuals) AML patients were enrolled in this study. AML patients were classified according to FAB classification Secondary and relapsed AML were judged by clinical history (12). Non APL patients in this study were treated with Cytarabin 100 to 200 mg/m2/day or Daunorubicin 60 mg/m2/day IV (N=51) and APL patients (N=8) were treated with Arsenic 45 mg/m 2/day (N=8). Clinical, morphologic, and cytogenetic data were outlined in Table 2. Similar to international reports, 60% AML patients who referred to B.M.T center during sampling time (n=103) were male (61 affected individuals) and 40% of them were female (42 individuals) but the average age of patients was 37.5 VS 67 in international reports. In patient’s group (n=59), the age of effected individuals ≤ 60 years was dominant (93%, 55 of 59) with a mean age of 39 (age of 16-86) Vs 67. Patients group showed a higher number of male patients (71%, 42 of 59). At sampling time, the range of blast percentage in patients’ bone marrow was 5%-100% (mean=72%) and hemoglobin range was 3-17.1 g/dL (average=8.7g/dL). Karyotyping reports indicated that 64.4% (38 of 59) of affected individuals had normal cytogenetic. We found one chromosomal aberration, 2 concomitant chromosomal aberration and complexity in karyotype in 13, 5, and 3 affected individuals respectively.

**Table 1:** DKN2B MCA primers, CDKN2B expression primers and GAPDH primers sequence. Tm of each primer was shown. MCA primers for CDKN2B was designed for promoter region flanking and expression primers were design exon exon junction

	**Tm**	**Sequence**	**Location/ Reference**
CDKN2B primers(MCA)	56.6° 53°	F: GGT TGG TTT TTT ATT TTG TTA GAG R: CCT AAA TTA CTT CTA AAA AAA AAC	2047-2070 2154-2177
CDKN2B primers (real-time)	58.4° 60.8°	F: TGG CCG GAG GTC ATG ATG R: GGG CAG CAT CAT GCA CCG	Exon exon junction
GAPDH primers	59.5° 56.4°	F: GAA GGT GAA GGT CGG AGT C R: GAA GAT GGT GAT GGG ATT TC	NM 002046

**Table 2:** Clinical characteristics of Iranian AML cohort (N _ 59 patients)

**Characteristics**	**Data**
Age, no. patients (%)	
Younger than 56 y	49 ( 83.1)
56 or older	10 (16.9)
Sex, no. patients (%)	
Female	17 (28.8)
Male	42 (71.2)
FAB classification, no. patients (%)	
M1	6 (10.2)
M2	18 (30.5)
M3	7 (11.9)
M4	9 (15.3)
M4E	1 (1.7)
M5	8 (13.6)
M6	3 (5.1)
Others	7 (11.9)
Specific cytogenetic features, no. patients (%)	
normal	40 (67.8)
abnormal	19 (32.2)
Cytogenetic risk category, no. patients (%)	
Favorable	9 (15.3)
Intermediate	40 (67.7)
Unfavorable	8 (13.6)
Not assigned	2 (3.4)
Median age, y (range)	36 (8 – 86)
Median WBC count,×10^9^/L (range)	12.87 (1.2 – 245.6)
Median marrow blasts, % (range)	72 (15 – 100)
Median platelet count, _ 10^9^/L (range)	40 (4 - 371)
Median hemoglobin level, g/dL (range)	8.5 (3 – 17.1)

favorable: inversion (16)/t (16;16)/del (16q), t (8;21), or t (15,17) with any additional abnormality; intermediate: _8, _Y, _6, del (12p), or normal karyotype; unfavorable: _5/del (5q), _7/del (7q), inversion (3q), abnormal 11q, t (6,9), t (9,22), abnormal 17p, or complex karyotype defined as more than 3 abnormality

**Figure 1:**
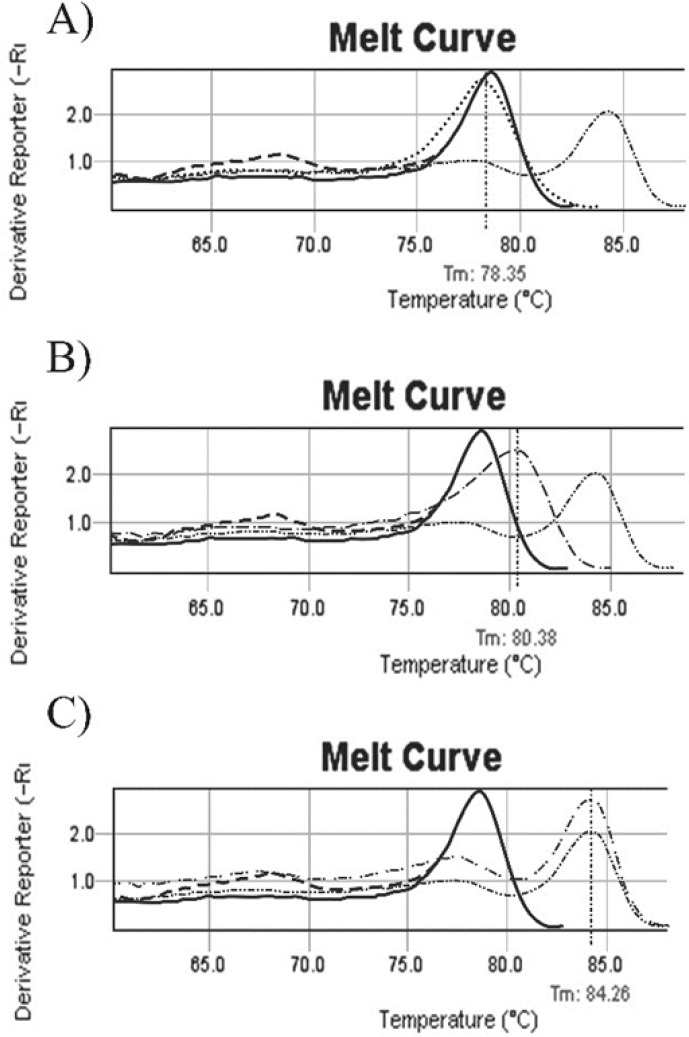
un-methylated, methylated and partial methylated melting curves. A: b014 melting curve (……) before treatment, Tm=78.35^ₒ^. Melt curve (….) is similar to normal individual control (---) and commercial un-methylated control (--). 1B: b035 patient melting curve (……) with tm= 80/38^ₒ^. Patient’s melt curves with shift to the right, is between normal individual control (---)/ commercial un-methylated control (----) and commercial methylated control (……..), this sample was considered as a partial methylated. 1C: Melting curve of b038 (…….), it is exactly similar to commercial methylated control curve (……..). Tm is 84.26^ₒ^. ---- Normal individual control, -- Commercial un methylated control, …… Commercial methylated control ---- Methylated sample, …… Partial methylated sample, ---- Un methylated sample after treatment, ……. Un methylated sample

**Figure 2:**
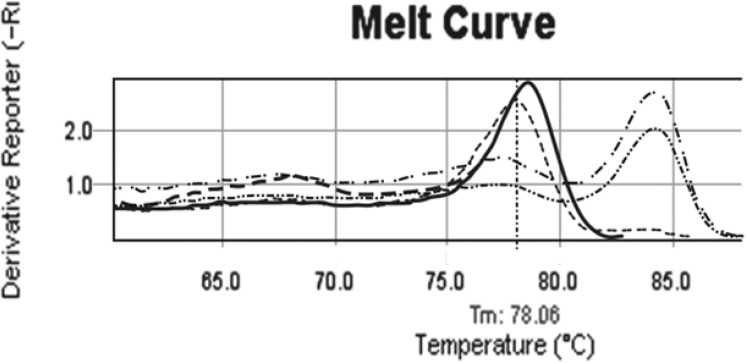
Comparison patient melting curve before and after treatment. b038 melting curve before treatment (….., tm= 84.26^ₒ^) is exactly as same as commercial methylated control. In D14 of treatment melting curve (----) is similar to un-methylated controls with tm= 78.06^ₒ^. In tis patients after treatment, methylation pattern of CDKN2B changed from full methylated (……..) to un-methylated pattern (…..). ---- Normal individual control, ---- Commercial un methylated control, …….. Commercial methylated control ……. Methylated sample, …… Partial methylated sample, …. Un methylated sample after treatment, ……. Un methylated sample

**Figure 3A:**
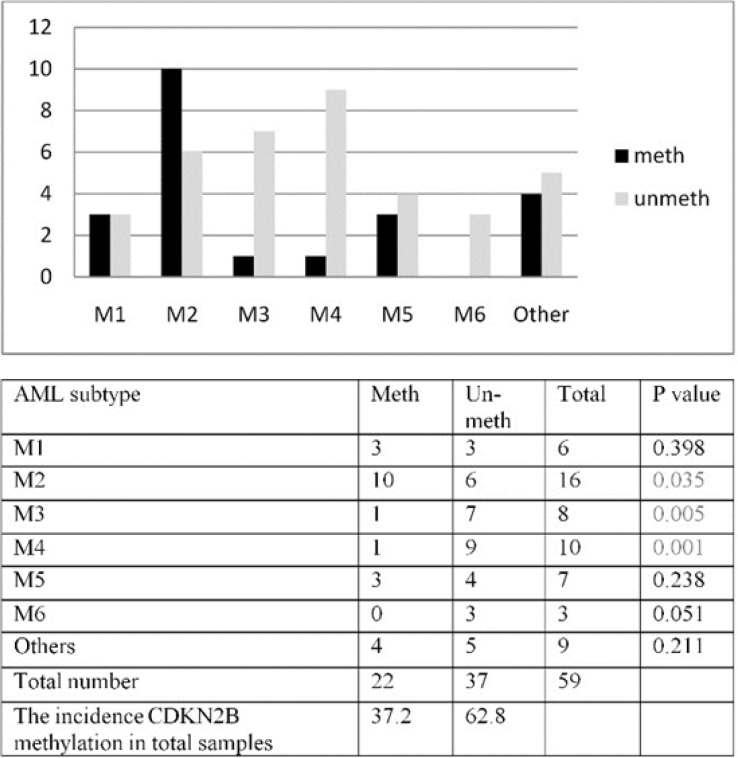
Affected patient’s number with Methylated / un-methylated CDKN2B in AML subgroups. B: Significant correlation between methylation and subtype s in M2, M3 and M4 (P=0.035,P=0.005, P=0.001 respectively) was found

**Figure 4:**
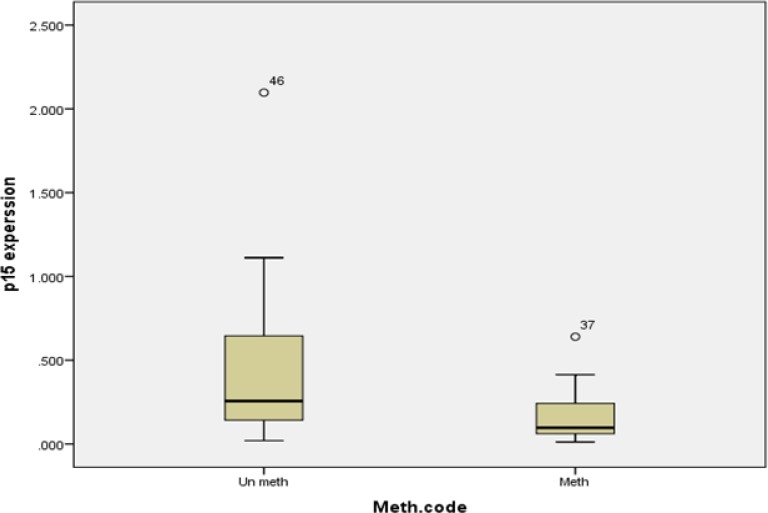
Box plot of expression level in methylated and un-methylated patients without considering subtype. As in curve has been shown there is significant correlation between CDKN2B gene expression and methylation (*P*=0.007

**Figure 5:**
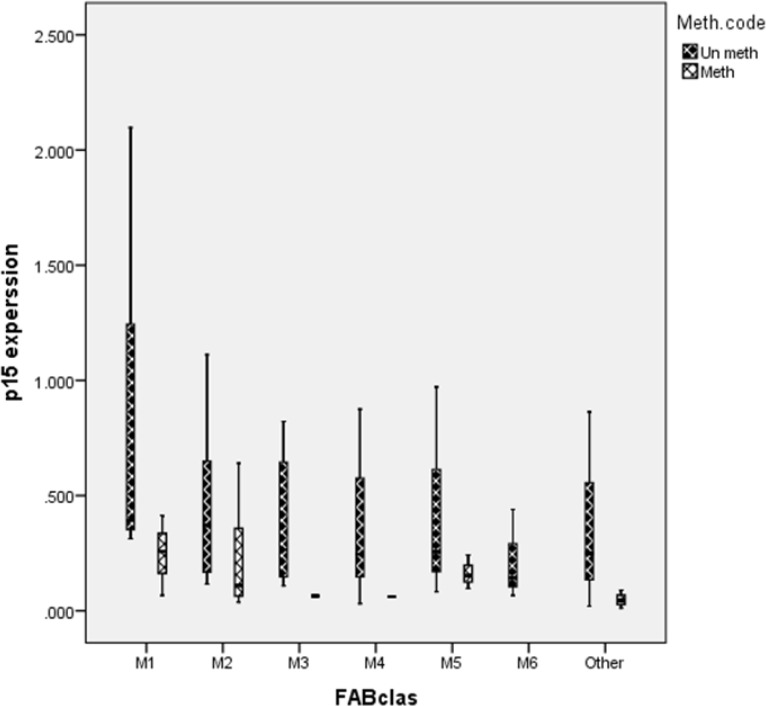
Box plot of expression in different AML subtypes. CDKN2B expression level didn’t show any significant difference between methylated and un methylated gene in AML patients (M1: *P*=0.3, M2: *P*=0.5, M3: *P*= 0.3, Other: *P*=0.1).

**Figure 6:**
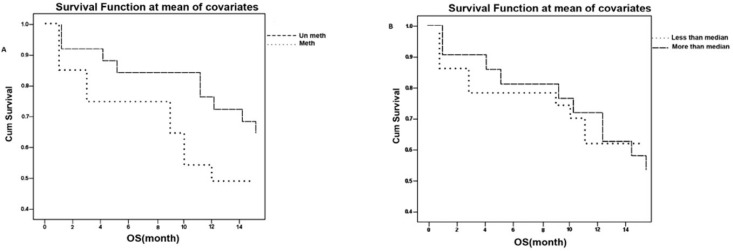
Survival curve in AML patients. A) Survival curve in AML patients according methylation shows that un-methylated patients have more survival in compare with methylated patients (*P*=0.043).

In this study, we revealed that 66% patients with age ≤60 year response to therapy (VS 70%-80% international reports). The rate of response to therapy in AML patients older than 60 year was 50% and this finding was similar to international reports. Our data illustrated significant correlation between survival and cytogenetic (*P*=0.032). 

*Promoter methylation of CDKN2B gene*

Promoter methylation of *CDKN2B* gene in all Samples was studied by Methylation Specific Melting Curve Analysis (MS-MCA) on bisulfite treated DNA. All melting curves were compared individually with methylated/ un-methylated commercial controls (EpiTect, QIAGEN) as shown in Figure 1.

Data illustrated that the incidence of aberrant hyper methylation of CDKN2B gene promoter region is 37.3% (22 of 59). We followed up 21 available enrolled patients, 7 of them indicated CDKN2B methylation. In the half of induction period, 5 of 7 methylated CDKN2B changed to un-methylated (Figure.2), 1 of 7 with full methylated CDKN2B changed to partial methylated. One of the patients in this group didn’t show any change in CDKN2B methylation in the half of induction period. Also this patient was resistant to treatment and expired less than one month. All un-methylated samples remained un-methylated during treatment.

In our patients, all the FAB subtypes included in the study except M6 showed *CDKN2B* methylation pattern. However, a higher frequency was encountered in patients with M2, M1 and M5 subtypes (62.5%, 50% and 42.9% respectively) as compared to M3 and M4 subtypes (28.5% and 22.2%) Figure.3A.

CDKN2B methylation was studied in different AML subtypes. The result of aberrant methylation of CDKN2B in different AML subtypes result showed higher incidence of methylated CDKN2B in M2 subtype (*P*=0.035), lower incidence of methylated CDKN2B in M3 (*P*=0.005) and M4 subtypes (*P*=0.001) Figure 3B This study revealed a significant correlation between hemoglobin level (Hb) and methylation in M1 and M5 subtypes (*P*=0.015, P=0.047 respectively).

Statistical analysis comparing CDKN2B methylation with other clinical factors revealed a significant correlation between methylation and bone marrow blast percentage (*P*= 0.022), white blood cell count (*P*=0.0123) in affected AMLs. 

*CDKN2B mRNA expression by real-time PCR*

*CDKN2B* mRNA expression was investigated in 58 of 59 patients. When normalized to normal mesenchymal stem cells, Patient’s mRNA *CDKN2B* expression range was 0.012-2.09 ± 0.39. Our data illustrated that 74% of enrolled patients (43 individuals) had more than two fold reduction (<0.5) in CDKN2B expression level and 26% of affected individuals (15 individuals) showed CDKN2B expression level more than 0.5. CDKN2B promoter methylation was analyzed in these 2 groups and the result showed 49% (21 of 43) of affected individuals with expression level less than 0.5 had methylated CDKN2B although, 13.3% (2 of 15) of enrolled patients with expression level more than 0.5 had methylated CDKN2B. CDKN2B expression level in 3 enrolled AML patients was high (13, 13, 5) and CDKN2B promoter was un-methylated. We observed the incidence of CDKN2B methylation in AML patients with expression level less than 0.1 is 64.7% (11 of 17) 

This study could exhibit a significant correlation between CDKN2B gene expression and methylation (*P*=0.007), but the correlation coefficient was low (Standardized Coefficients =0.360). CDKN2B expression in patients with methylated CDK2B gene and patients with un-methylated CDKN2B gene has been shown in Figure.4

Besides, CDKN2B gene expression level in different AML subtypes didn’t reveal any significant correlation between expression level and methylation in different subtypes (M1: *P*=0.3, M2: *P*=0.5, M5: *P*=0.4, others: *P*=0.2). Methylated CDKN2B gene expression and un-methylated CDKN2B gene expression in different AML are presented in Figure.5.

*Survival Analysis*

In order to investigate the patients’ survival we managed to follow 48 patients up to 14 months. Our data indicated that 37.5% of available patients (18 of 48) had survival less than 1 year and 62.5% (30 of 48) of available affected individuals had survival more than 1 year. Our study illustrated that patients with CDKN2B un-methylated gene had more survival (*P*=0.043) compared to patients with CDKN2B un-methylated gen. Figure 6A. 

The survival was investigated in patients classified according to median of expression level and the data didn’t indicate a significant correlation between survival and expression level (*P*=0.93). Figure 6B. 

However, we couldn’t indicate any significant correlation between aberrant methylation of CDKN2B and response to therapy (*P*=0.18).

## Discussion

In order to investigate using methylated CDKN2B gene as a diagnostic or prognostic biomarker, methylation and expression of CDKN2B analysis was performed in 59 AML patients’ sample. According to the American Cancer Society, the average age of AML patients is 67 years and AML is slightly more common in males (60%) than in females (40%). In the study cohort, the male were more than the female (70% VS 30%) and enrolled patients were younger than international reports (39 VS 67). In order to confirm this issue that the age of Iranian AML patients is younger than international reports, we need to more specific studies. Also, more specific ethical and cultural studies were recommended to figure out the reason that the AML affected male are more than the female. Although some previous studies have showed the effect of economic and insurance with this issue (17, 18), but more specific studies about insurance effect on this issue are recommend.

 Some previous studies such as Prisler *et al*. Raji *et al*. and Denberg *et al*. showed the importance of CDKN2B methylation as a biomarker in diagnosis, response to treatment and secondary hematology disease. Furthermore, new studies have indicated that epigenetic targeting and personalized is a new approach for AML (19-22). In this study, we have analyzed *CDKN2B* methylation and expression in a cohort of Iranian AML patients and available enrolled patients were followed up more than one year. Frequency of CDKN2B promoter methylation in all AML subtypes (%37.3) were similar to Galm *et al*. findings (%32) (5), but it was different from Shimamoto (51%) (23) finding. There have been a lot of variations in reported frequency of *CDKN2B* promoter methylation in different AML studies varying from 31% to 93% (5, 24-26). The variations between different studies may be attributed by the different promoter regions studied, number of CpG analyzed, sensitivity of techniques and patient numbers included in each study (27-29).

We investigated the correlation between CDKN2B methylation and different clinical features of our patients. Our analysis revealed a significant correlation between CDKN2B and bone marrow blast percentage (*P*=0.022) as reported previously (30). Additionally, our study could prove more correlation between clinical and sub clinical in some of subtypes of AML. For example, our data showed a significant correlation between methylation and Hb level in M1 (*P*=0.015) and M5 (*P*=0.047), although the biological significance of this finding should be investigated in further studies. Our results showed higher incidence of methylated CDKN2B in M2 subtype (*P*=0.035) and lower methylated CDKN2B in M3 (*P*=0.005) and M4 subtypes (*P*=0.001) Figure.3 This result was different from Christiansen *et al* and Wong *et al.* and similar to El-Shakankiry *et al*. finding. Christiansen reported that incidence of methylation of CDKN2B is less in AML M5 and Wong reported that frequency of methylation in M2 and M4 is higher than the other subtypes and he showed CDKN2B methylation status highly correlated with morphologic disease stage (25, 27, 28) but further studies need to find the correlation between FAB classification and CDKN2B methylation. The differences between observed results in different studies can be caused by differences in sensitivity or specificity of techniques and number of patients being included (26-29). Our data revealed a significant correlation between CDKN2B promoter methylation and gene expression (*P*=0.007), as previously reported by Preisler and Denberg (20-22). But, the correlation coefficient was low which indicates that the *CDKN2B* mRNA expression level is controlled by other cellular mechanisms in addition to methylation. Expression of CDKN2B gene has been reported to be controlled by *TGF* *beta*, (31) and histone modification (32). 

Our results did not illustrate a significant correlation between response to treatment and methylation (*P*=0.18). But some studies showed that CDKN2B has been used as a prognostic biomarker and a marker to show relapse (21-23, 28). According to interview with patients, therapeutic plane was not standard and social and economic limitations were some reasons to avoid standard therapy and might cause our study didn’t reveal significant correlation between methylation and remission, but more studies need to confirm it. 

In this study, survival indicated a significant correlation between methylation (*P*=0.043) (Figure 4) and these result are the same as those of Denberg *et al.* and Wnog *et al.* studies (21, 28). Denberg in 2011 reported a significant correlation between survival and global promoter hypo-methylation in this gene. He found that patients with methylated CDKN2B promoter has more survival compared to patients with un-methylated gene. This finding was reverse to his previous finding. According to Denberg, these discrepancies are related to number and location of CpG Island methylation. Because some of CpG Islands are target for specific poly comb genes and these islands are necessary for epigenetic machinery (21, 22). 

Overall, our data showed that the Iranian AML individuals are younger than international reports (39 VS 67) (Table 1). Moreover, in spite of 37.2% incidence of CDKN2B methylation, we found 2 fold reductions in wide majority of enrolled patients and it confirm that methylation is not the only pathway for CDKN2B expression control. Although, we could prove that methylated CDKN2B is a biomarker in following up of patients. In order to increase survival patients, we can recommend to oncologists that consider change of epigenetic pattern using drug that reverse aberrant hyper methylation, such as 5-aza-2′-deoxycytidine and 5-azacytidine, in patients therapy plane. But further studies in this matter warranted determining whether demethylating agent could sensitize the resistant cell to conventional cytosine drug in AML or not.

## References

[1] Girish Babu KL, Doddamani G M, Mathew J, Jagadeesh K N, Ramanaik Naik LR (2015). Pediatric leukemia. J. Pediatr. Dent..

[2] Jaenisch R, Bird A (2003). Epigenetic regulation of gene expression: how the genome integrates intrinsic and environmental signals. Nature genet..

[3] Oki Y, Issa JP (2010). Epigenetic mechanisms in AML - a target for therapy. Cancer treat. Res..

[4] Rodrigues EF, Santos-Reboucas CB, Goncalves Pimentel MM, Mencalha AL, Dobbin J, Da Costa ES, Fernandez CD, Bouzas LF, Abdelhay E, Fernandez TD (2010). Epigenetic alterations of p15(INK4B) and p16(INK4A) genes in pediatric primary myelodysplastic syndrome. Leuk. Lymphoma..

[5] Galm O, Wilop S, Luders C, Jost E, Gehbauer G, Herman JG, Oseika R (2005). Clinical implications of aberrant DNA methylation patterns in acute myelogenous leukemia. Ann. Hematol..

[6] Ekmekci CG, Gutierrez MI, Siraj AK, Ozbek U, Bhatia K (2004). Aberrant methylation of multiple tumor suppressor genes in acute myeloid leukemia. Am. J. hematol..

[7] Alvarez S, Suela J, Valencia A, Fernandez A, Wunderlich M, Agirre X, Prósper F, osé Martín-Subero L, Maiques A, Acquadro F, Perales SR, Calasanz MJ, Roman-Gómez J, Cigudosa JC (2010). DNA methylation profiles and their relationship with cytogenetic status in adult acute myeloid leukemia. PlOS One.

[8] Perrone F, Tamborini E, Dagrada GP, Colombo F, Bonadiman L, Albertini V, Lagonigro MS, Gabanti E, Caramuta S, Greco A, Torre GD, Gronchi A, Pierotti MA, Silvana Pilotti (2005). 9p21 locus analysis in high-risk gastrointestinal stromal tumors characterized for c-kit and platelet-derived growth factor receptor alpha gene alterations. Cancer.

[9] Fares J, Koller R, Humeniuk R, Wolff L, Bies J (2012). The tumor suppressor p15Ink4b regulates the differentiation and maturation of conventional dendritic cells. Blood.

[10] Drexler HG (1998). Review of alterations of the cyclin-dependent kinase inhibitor INK4 family genes p15, p16, p18 and p19 in human leukemia-lymphoma cells. Leukemia.

[11] Genomic, Epigenomic Landscapes of Adult De Novo Acute Myeloid Leukemia (2013). N Engl. J. Med..

[12] Bennett JM, Catovsky D, Daniel MT, Flandrin G, Galton DA, Gralnick HR, Sultan C (1976). Proposals for the classification of the acute leukaemias French-American-British (FAB) co-operative group. Br. J. Haematol..

[13] Brennan DC, Lewis JP (1980). Remission-induction regimens in acute nonlymphocytic leukemia. West. J. Med..

[14] Guldberg P, Worm J, Gronbaek K (2002). Profiling DNA methylation by melting analysis. Methods (San Diego, Calif).

[15] Vaerman JL, Saussoy P, Ingargiola I (2004). Evaluation of real-time PCR data. J. Biol. Regul. Homeost. Agents..

[16] Livak KJ, Schmittgen TD (2001). Analysis of relative gene expression data using real-time quantitative PCR and the 2(-Delta Delta C(T)) Method. Methods (San Diego, Calif).

[17] Nerich V, Lioure B, Rave M, Recher C, Pigneux A, Witz B, Escoffre-Barbe M, Moles MP, Jourdan E, Cahn JY, Woronoff-Lemsi MC (2011). Induction-related cost of patients with acute myeloid leukaemia in France. Int. J. Clin. Pharm..

[18] Bradley CJ, Dahman B, Jin Y, Shickle LM, Ginder GD (2011). Acute myeloid leukemia: how the uninsured fare. Cancer.

[19] Raj K (2007). CDKN2B methylation status and isolated chromosome 7 abnormalities predict responses to treatment with 5-azacytidine. Leukemia.

[20] Prisler HD (2001). P15INK4B gene methylation and expression in normal, myelodysplastic, and acute myelogenous leukemia cells and in the marrow cells of cured lymphoma patients. Leukemia.

[21] Deneberg S, Grovdal M, Karimi M, Jansson M, Nahi H, Corbacioglu A, Gaidzik V, Dohner K, Paul C, Ekstrom TJ (2010). Gene-specific and global methylation patterns predict outcome in patients with acute myeloid leukemia. Leukemia.

[22] Deneberg S, Guardiola P, Lennartsson A, QuY, Gaidzik V, Blanchet O, Karimi M, Bengtzen S, Nahi H, Uggla B, TidefeltU, HöglundM, Paul C, Ekwall K, Döhner K, Lehmann S (2011). Prognostic DNAmethylation patterns in cytogenetically normal acute myeloid leukemia are predefined by stem cell chromatin marks. Blood.

[23] Shimamoto T, Ohyashiki JH, Ohyashiki K (2005). Methylation of p15(INK4b) and E-cadherin genes is independently correlated with poor prognosis in acute myeloid leukemia. Leukemia Res..

[24] Aggerholm A, Guldberg P, Hokland M, Hokland P (1999). Extensive intra- and interindividual heterogeneity of p15INK4B methylation in acute myeloid leukemia. Cancer Res..

[25] Christiansen DH, Andersen MK, Pedersen-Bjergaard J (2003). Methylation of p15INK4B is common, is associated with deletion of genes on chromosome arm 7q and predicts a poor prognosis in therapy-related myelodysplasia and acute myeloid leukemia. Leukemia.

[26] Das-Gupta EP, Russell NH (2000). Anticorresponding p15 promoter methylation and microsatellite instability in acute myeloblastic leukemia. Blood.

[27] El-Shakankiry NH, Mossallam GI (2006). p15 (INK4B) and E-cadherin CpG island methylation is frequent in Egyptian acute myeloid leukemia. J. Egypt. Natl. Canc. Inst..

[28] Wong IH, Ng MH, Huang DP, Lee JC (2000). Aberrant p15 promoter methylation in adult and childhood acute leukemias of nearly all morphologic subtypes: potential prognostic implications. Blood.

[29] Toyota M, Ahuja N, Suzuki H, Itoh F, Ohe-Toyota M, Imai K, beylin SB, Issa JP (1999). Aberrant methylation in gastric cancer associated with the CpG island methylator phenotype. Cancer Res..

[30] Kim M, Oh B, Kim SY, Park HK, Hwang SM, Kim TY, She CJ, Yang I, Yoon SS, Yoon JH, Lee DS (2010). p15INK4b methylation correlates with thrombocytopenia, blast percentage, and survival in myelodysplastic syndromes in a dose dependent manner: quantitation using pyrosequencing study. Leukemia Res..

[31] Hannon GJ, Beach D (1994). p15INK4B is a potential effector of TGF-beta-induced cell cycle arrest. Nature.

[32] Hopfer O, Komor M, Koehler IS, Schulze M, Hoelzer D, Thiel E, Hafmann WK (2007). DNA methylation profiling of myelodysplastic syndrome hematopoietic progenitor cells during in-vitro lineage-specific differentiation. Exp. Hematol..

